# General practitioners’ views on use of patient reported outcome measures in primary care: a cross-sectional survey and qualitative study

**DOI:** 10.1186/s12875-019-1077-6

**Published:** 2020-01-24

**Authors:** Grace M. Turner, Ian Litchfield, Sam Finnikin, Olalekan Lee Aiyegbusi, Melanie Calvert

**Affiliations:** 1 Institute of Applied Health Research, University of Birmingham, B15 2TT, Birmingham, UK; 2 Centre for Patient Reported Outcomes Research, University of Birmingham, B15 2TT, Birmingham, UK; 3 NIHR Birmingham Biomedical Research Centre, University Hospitals Birmingham NHS Foundation Trust and University of Birmingham, Birmingham, UK

**Keywords:** Patient reported outcome measures (PROMs), Primary care, General practitioners, Qualitative, Survey

## Abstract

**Background:**

Patient reported outcome measures (PROMs) are increasingly used to assess impact of disease and treatment on quality of life and symptoms; however, their use in primary care is fragmented.

We aimed to understand how PROMs are currently being used in primary care, the barriers and facilitators of this use and if appropriate how it might be optimised.

**Methods:**

Cross-sectional survey and semi-structured interviews among general practitioners (GPs) in England. GPs’ opinions were explored using an electronic, self-completed questionnaire disseminated to 100 GPs via an online doctors’ community and semi-structured qualitative interviews with 25 GPs.

**Results:**

Most GPs surveyed (77/100; 77%) reported using one or more PROM, primarily to aid clinical management (*n* = 66) or as screening/diagnostic tools (*n* = 62).

Qualitative interviews highlighted challenges in identifying and selecting PROMs; however, some GPs valued PROMs for shared decision making and to direct patient discussions. The interviews identified key barriers to PROM use including: time constraints; insufficient knowledge; lack of integration into clinical systems; and PROMs being mandated without consultation or explanation. Evidence of the benefit of PROMs is required to promote uptake and use of PROMs in primary care.

**Conclusion:**

Implementation of PROMs in primary care requires integration with clinical systems, a bottom-up approach to PROM selection and system design involving meaningful consultation with patients and primary care clinicians and training/support for use.

## Introduction

Patient Reported Outcome Measures (PROMs) are increasingly used to measure patients’ own experience of their health, such as symptoms, mobility, mental health and social function [1]. Capturing PROMs alongside traditional clinical outcomes can offer a range of benefits: at an individual patient level PROMs can facilitate communication, identification of problems and help tailor care to needs [2–4]; at aggregate level these data can be used as real world evidence of treatment effectiveness and can be used for audit/benchmarking purposes and to assess service delivery/reconfiguration [5].

In the United Kingdom, there are a few cases of PROMs use being implemented in primary care. For example, completion of the Patient Health Questionnaire-9, a depression screening PROM, was incentivised between 2006 and 2013 as part of the pay-for-performance quality and outcomes framework (QOF) [6]. In addition, completion of the Oxford Knee Score is a requirement of a referral pathway for knee replacement [7]. However, beyond mandated use, anecdotal evidence suggests PROM use in primary care is fragmented and ad hoc. The heterogeneous nature of primary care, such as the diverse range of conditions, symptoms and outcomes, may present challenges for PROM selection and use in this setting [8]. We aimed to understand the current use of PROMs in primary care, barriers and facilitators, and if appropriate how their use might be optimised.

## Method

The study was a mixed methods design comprising a survey and qualitative interviews. The study was approved by the University of Birmingham the Science, Technology, Engineering and Mathematics Ethical Review Committee (Reference ERN_16-0568S).

### Online survey

An electronic, self-completed questionnaire was disseminated to general practitioners (GPs) in England via an online doctors’ community (www.doctors.net.uk) in December 2017. A sample size of 100 was pre-specified based on funding limitations. The survey explored GPs’ current use of PROMs; barriers to PROM use; and how PROMs could ideally be used in primary care (Tables 2 and 3) and would take an estimated ten minutes to complete.

### Qualitative study

GPs were recruited from amongst those that had completed the online survey and by using convenience sampling of known contacts and snowballing [9]. Semi-structured interviews were conducted by telephone. All interviews were conducted by IL, a Research Fellow employed by the University of Birmingham. The interviewer did not have a relationship with any of the participants. A semi-structured topic guide was developed based on the online survey findings. Use of the topic guide ensured key topics were consistently covered including: how PROMs were used, by whom, in which circumstances, and barriers/ facilitators including influences of patient, clinician, and system factors (see Additional file 1). Interviews were conducted between June and September 2018. Data was collected and analysed until data saturation was reached [10].

Interviews were audio recorded using a digital recorder, and recordings were transcribed verbatim by a professional transcription service. NVivo v12 was used to manage, sort, code and organise the transcribed data.

### Data analysis

Quantitative survey data was summarised using descriptive statistics and free text comments were categorised into themes. Qualitative interview data was thematically analysed [11]. Transcripts were read several times to enable the researcher to familiarise themselves with the data. IL (experienced qualitative researcher) coded all transcripts and OLA independently coded a subset (10%). Open coding was applied to the transcripts, codes were then reviewed, organised in categories and, after being refined, overarching themes. The final analysis and interpretation was discussed with the wider team.

## Results

### Online survey

The online survey was completed by 100 GPs across England (Table 1). Seventy seven GPs reported using one or more PROM; however, 17% (38/224) of the measures reported were not PROMs (for example cardiovascular risk scores). The majority of PROMs reported were for mental health (*n* = 85 PROMs), urology (*n* = 37 PROMs), sleep apnoea (*n* = 25 PROMs) or orthopaedics (*n* = 19 PROMs). A description of the most commonly used patient-reported outcome measures in primary care can be found in the Additional file 1: Table S2.

**Table 1:** Characteristics of GPs completing the survey (*n* = 100)

		Number
Years GMC GP Register	≤10	35
	11–20	44
	≥21	21
Primary role	Salaried	25
	Locum	16
	Partner	62
	Academic	4
	Commissioner	4
	Other	1
Full/ part time	Full time	64
	Part time	36
Rurality of GP practice	Urban	38
	Suburban	43
	Rural	13
	Both	6
Region of GP practice	East of England	9
	East Midlands	9
	London	15
	North East	6
	North West	11
	South Central	10
	South East Coast	8
	South West	11
	West Midlands	11
	Yorkshire and Humber	10

*GP* General Practitioner, *GMC* General Medical Council

The most common reasons for PROM use were to aid clinical management (*n* = 66) or as a screening/ diagnostic tool (*n* = 61) (Table 2). GPs most frequently accessed PROMs through clinical systems (*n* = 56), clinical templates (*n* = 49) or online (*n* = 47). PROMs were usually completed during the consultation (*n* = 72), using paper (*n* = 68) or GP administered interviews (*n* = 51), and were reviewed by GPs (*n* = 84). The main barriers reported for PROM use were time constraints (*n* = 77) and being mandated to use without consultation or explanation (*n* = 55). When asked “how do you think your patients feel about completing PROMs?”, most GPs responses were positive or neutral (n = 47 and *n* = 31, respectively).

**Table 2:** Survey questions about GPs current use of PROMs (*n* = 100)

Question	Multiple choice answer	Number
What do you currently use PROMs for?^a^	To aid clinical management	66
	As a screening/diagnostic tool	61
	Facilitate shared-decision making	48
	To improve efficiency of consultation	45
	Facilitate communication with patients	42
	For Chronic disease monitoring	38
	To support personalised care planning and self-management	31
	Facilitate communication across different healthcare sector	28
	Monitor performance	25
	For triage	14
	To monitor safety/adverse events	12
	Facilitate communication between patients and family members/ carers	11
	Reward performance	10
	Research	5
	Other	2
	None – I don’t currently use PROMs	19
How do you access PROMs?^a^	Through clinical systems	56
	Embedded within clinical templates	49
	Online	47
	Paper	29
	Other	3
	N/A – I don’t access PROMs	11
When do your patients complete PROMs?^a^	During the consultation: face-to-face	72
	Prior to consultation: at home at the request of a healthcare professional	22
	Prior to consultation: in the waiting room	12
	During the consultation: telephone	9
	Prior to consultation: at home, instigated by the patient	2
	Other	20
	N/A – my patients don’t currently complete PROMs	17
How do your patients complete PROMs?^a^	Paper	68
	Interview: in person (doctor)	51
	Interview: in person (nurse)	18
	Interview: over the phone (doctor)	13
	Interview: over the phone (nurse)	6
	Online	5
	Completed by a proxy on behalf of patients	5
	Through an app	3
	N/A – my patients don’t currently complete PROMs	15
Who reviews the results of PROMs?^a^	Doctor	84
	Nurse	17
	Other	3
	N/A – my patients don’t currently complete PROMs	15
What do you think are the main barriers to use of PROMs?^b^	Time constraints	77
	Mandated to complete	55
	Sufficient understanding without PROMS	32
	Patients dislike questionnaires	31
	Uncertainty about reliability	27
	Perceived as cost-cutting	21
	Constrain doctor-patient relationship	18
	Lack of integration into clinical systems	13
	Feels uncomfortable	11
	Number of measures	10
	Lack of confidence in interpreting	8
	Other	4
	None	1
How do you think your patients feel about completing PROMs?^c^	Negative (e.g. chore, tick box, not interested)	22
	Positive (e.g. happy to complete, time saving, longitudinal outcomes)	47
	Neutral	31

^a^tick all that apply; ^b^rank top 3; ^c^ free text categorised into themes

*N/A* Not Applicable, *PROMs* Patient Reported Outcome Measures

Similar to current PROM use, the most common areas GPs considered PROMs could provide the most benefit were to aid clinical management (*n* = 66), as a screening/ diagnostic tool (*n* = 62) or facilitate shared-decision making (*n* = 60) (Table 3). GPs would prefer to access PROMs through clinical templates (*n* = 65) or clinical systems (*n* = 63) and considered patients would prefer to complete PROMs during the consultation (*n* = 34). The preferred format for patients to complete PROMs was considered to be paper (*n* = 43) or online (*n* = 21), and the preferred format for GPs recording PROM results was electronic: as part of the electronic health record (*n* = 78). GPs viewed that doctors would be the most appropriate people to interpret PROM results (n = 78). The patient groups/conditions where GPs considered there would be the most benefit from completing PROMs was mental health (*n* = 20), all/ most patients (*n* = 15) and patients with chronic conditions (*n* = 14). GPs considered PROMs should be collected for chronic disease monitoring annually (*n* = 36) or as clinically indicated (*n* = 27). Most GPs felt development of PROM systems should be designed to meet clinician/ patient needs (*n* = 68) rather than designed primarily for audit, benchmarking or commissioning (*n* = 3). Integrated clinical systems (*n* = 29), more time (*n* = 12) and easy access (*n* = 12) were the most common features GPs reported that could facilitate/ support the use of PROMs in primary care.

**Table 3:** Survey questions about how PROMs could ideally be used in primary care (*n* = 100)

Question	Multiple choice answer	Number
Where do you think PROMs could provide the most benefit?^a^	To aid clinical management	66
	As a screening/diagnostic tool	62
	Facilitate shared-decision making	60
	Facilitate communication with patients	43
	To support personalised care planning and self-management	39
	For chronic disease monitoring	34
	To improve efficiency of consultation	31
	Monitor performance	31
	Facilitate communication across different healthcare sectors	22
	Facilitate communication between patients and family members/ carers	17
	For triage	16
	Research	16
	To monitor safety/ adverse events	15
	Reward performance	7
	None – I don’t think PROMs provide a benefit	5
How would you prefer to access PROMs?^a^	Embedded within clinical templates	65
	Through clinical systems	63
	Online	27
	Paper	16
	Other	1
	NA – I don’t want to access PROMs	4
When do you think patients would prefer to complete PROMs?	During the consultation: face-to-face	34
	Prior to consultation: at home at the request of a healthcare professional	25
	Prior to consultation: in the waiting room	20
	Prior to consultation: at home, instigated by the patient	11
	During the consultation: telephone	1
	Other	5
	NA – I don’t think my patients will complete PROMs	4
What would the best format be for patients to complete PROMs?	Paper	43
	Online	21
	Interview: in person (doctor)	16
	Through an app	8
	Interview: in person (nurse)	3
	Interview: over the phone (doctor)	1
	Interview: over the phone (nurse)	1
	Other	4
	N/A – I don’t think my patients should complete PROMs	3
Who do you think should ideally review the results of PROMs?	Doctors	78
	Nurses	7
	Other (please specify)	15
How frequently do you think PROMs should be used for chronic disease monitoring?	Annually	36
	As prescribed	27
	Monthly	13
	Whenever the patient choses	9
	Prior to every consultation	8
	I don’t think there is a role for PROMs in chronic disease monitoring	7
What format for recording results would you prefer for PROMs?	Electronic (as part of the electronic health record)	78
	Paper	11
	Electronic (patient portal)	10
	Telephone	1
Do you think development of PROM systems should be:	Designed to meet clinician/patient needs (Bottom up approach)	68
	Try to meet both objectives	29
	Designed primarily for audit, benchmarking or commissioning (Top down approach)	3
Which of your patients do you think would benefit the most from completing PROMs?^b^	Mental health patients	20
	All / most patients	15
	Patients with chronic conditions	14
	Unsure	8
	Patients interested in PROMs	8
	None/very few patients	6
	Patients who struggle to communicate/articulate symptoms	6
	Younger patients	4
	Educated/literate patients	3
	Cognitive impairment patients	2
	Other	35
Is there anything that facilitates/supports the use of PROMs in primary care?^b^	Integrated clinical systems	29
	More Time	12
	Easy access	12
	Unsure	9
	Ease of use	9
	Training, knowledge, experience	7
	None	21
	Other	22

^a^tick all that apply; ^b^ free text categorised into themes

*N/A* Not Applicable, *PROMs* Patient Reported Outcome Measures

### Qualitative study

The final sample comprised 25 GPs, participant characteristics are detailed in Table 4 and Additional file 1: Table S1. Interviews lasted between 18 and 59 min.

**Table 4:** Participant characteristics (*n* = 25)

Variable		Number (%)
Sex	Male	14 (58)
	Female	11 (42)
Years qualified	< 5	3 (12)
	5–10	7 (28)
	11–20	10 (40)
	21–30	3 (12)
	> 30	2 (8)
Region	North East	5 (20)
	North West	4 (16)
	East Midlands	1 (4)
	West Midlands	5 (20)
	South	3 (12)
	South East	6 (24)
	South West	1 (4)

### Current use of PROMs

PROMs were considered useful to aid shared decision making, the ability of PROMs to provide an objective measure that could be used in subsequent discussions of treatment was described.

*“It does help direct the discussion regarding future management, especially the mental health patients because it allows them to objectively score how they feel and what’s going on, and allows me to help discuss treatment options with them.” GP12*

By completing PROMs with patients, one GP described how they facilitated the discussion with a patient about their symptoms.

*“I have sometimes filled in the questionnaires with the patients, and actually I see the value of that because we actually get a better picture with the discussion around filling the form with the patient as opposed to just getting a figure just attached to the referral letter.” GP13*

### Facilitators to PROM use

GPs knowledge and understanding of the value/benefits of PROMs to their clinical practice was an important factor in their use with some GPs considering that better communication of the evidence-base for PROMs could be an important driver for their take-up.

*“For me I am quite evidence based personally, and if someone was to show me like you’re doing, if I’m the outlier and most GPs love PROMs and I would actually be thinking hand on I’m the outlier here, actually maybe I’ll just get more on board. If there was a study saying this particular PROM if they said PHQ-9 shortened a ten minute consultation down to five minutes, improves on patient outcomes, reduces re-attendance rates, improve compliance to medications, then I would say right we’ve got to get on board and do that.” GP18*

Related to the reliability of the evidence base one GP described how they were more likely to use PROMs if they heard about them from multiple sources, corroborating perceptions of their utility.

*“ … I am unlikely to go and start using some new coeliac disease PROM when I have just been to a talk from a private gastroenterologist or something like that. I am more likely to use something that is appearing to me in lots of different areas of my CPD or medical education. So if I might see a paper about it, and then I might hear a colleague talking about it, and then I might see something on GP Notebook or something like that. So you’re getting over exposed to it, and then try it out and see how well it resonates, and how useful it is and how quick and easy to remember it is.” GP24*

### Barriers to PROM use

Some GPs were unconvinced of the benefits of PROMs instead placing the onus on clinical data. This appeared at least in part due to concerns of the reliability of patients whose responses might be influenced by attempts to manipulate the output for their own purposes.

*“ … sometimes the patient can fill them in with what they think the clinician might want them to say rather than what they actually feel. So sometimes patients can underplay their symptoms, and equally sometimes patients can overplay their symptoms if there might be some perhaps secondary gain for them in terms of certification from work or whether they want some help with some other part of their care. So I think they can potentially be a bit skewed by that.” GP19*

Though the use of PROMs can be encouraged at a policy level, this top-down approach for mandatory PROM completion was objected to by GPs and did not convince them of the value of PROMs. For example one GP felt obliged to use a PROM solely due to the financial incentive offered by the National Health Service (NHS) pay-for-performance scheme Quality Outline Framework (QOF) in the absence of any clinical value. This is supported by its presence on the dashboard of their clinical management software.

*“so the big one is PHQ-9, it’s pushed very hard and for example with people with chronic diseases as well it flags up in the QOF box on EMIS. But in reality it’s irrelevant to assisting you that much in terms of referral and management, so there’s no point in doing it.” GP18*

Lack of time in the consultation to complete, analyse and integrate an additional source of information was described and one GP felt this was a barrier to their routine use.

*“In a pressurised rushing surgery and you’ve only got ten minutes the person usually would need at least 20 minutes to solve their issues, and if you were to include a questionnaire on top of that you would be definitely talking about 30 minutes at least, and you can’t afford to be doing that on a regular basis. You can do it as a one off thing and then you have an idea, but you would be pressurised to just do things quickly...” GP2*

Lack of integration with clinical systems was also identified as a potential barrier; in some cases PROMs were integrated in clinical systems but they were not easy to use.

*“So we do have some which are integrated into the system, but they are not quite integrated enough to be user friendly, … I’m actually thinking here of the dementia screening test which isn’t really a PROM I guess, but you have to input the data and then the score is added up wrong because the template is set up wrong, so you end up having to override it and do it yourself anyway which makes it a bit of a waste of time it being integrated, and I think sometimes with the clunky way that clinical computer systems sometimes work it be difficult during the consultation to use that tool there and then. So I think there could be better integration, it could be more fluid, and I suppose depends on which clinical system is used, which PROMs are used locally and how easy they are to integrate into the system, because I guess some are going to be more objective than others.”* GP19

Though large numbers of PROMs have been produced there appeared no systematic method of communicating their identity and availability to practice staff. For one GP the subsequent lack of awareness significantly reduced their uptake.

*“To make me want to use a PROM first of all I would have to hear about it, and that is the main problem that we wouldn’t hear about it and therefore people get them on committees as mandatory things to put down on referrals. That happens because we don’t hear … ” GP20*

## Discussion

### Summary

Most GPs surveyed reported using one or more PROM, primarily to aid clinical management or as screening/diagnostic tools. Qualitative interviews highlighted challenges in identifying and selecting PROMs; however, some GPs valued PROMs for shared decision making and to direct patient discussions. Key barriers to PROM use included: time constraints; insufficient knowledge; lack of integration into clinical systems; and PROMs being mandated without consultation or explanation. Understanding of the value/benefits of PROMs to clinical practice and hearing about PROMs from multiple different sources were facilitators for PROM use. Whilst PROMs may offer a range of benefits, a more systematic awareness of the elements affecting their successful implementation is needed [12]. In this way the co-design of systems and processes incorporating PROMs and the development of a coherent evidence base can successfully underpin their uptake and roll out.

### Strengths and limitations

Key strengths of our study include the mixed methods design and national sampling of GPs across England. However, the survey sample size was restricted to 100 GPs due to funding constraints. Similarly we recognise the importance of patient perspectives on the use of PROMs but it is beyond the scope of this study. Only members of the online doctors’ community could access the survey which may limit generalisability of survey results. Furthermore, 17% of measures reported in the survey were not PROMs; therefore, some GPs survey responses may be in reference to risk scores. We interviewed GPs from across England with a balance of genders and a range of experience and though broad and descriptive in nature this preliminary analysis has allowed us to begin to understand the nature of the influences on PROMs adoption.

### Comparison with existing literature

We found that PROMs were considered to impair communication with patients by some GPs. Similarly in other studies, GPs have reported that PROMs can disrupt the flow of consultations through ‘mechanistic’ questions which ‘trivialise’ patients’ emotions [13–15]. In contrast, patients have reported that they found PROMs facilitated communication with GPs and were helpful prompts to share issues [16]. In addition, PROMs completion can change how patients think about their condition; therefore, are more than just modes of information collection [17]. A recent (2018) systematic review of reviews exploring facilitators and barriers to implementing PROMs in organisations delivering health related services highlighted the important of clinicians valuing PROMs and understanding their validity [18]. Therefore, effective implementation of PROMs in requires time and resources invested appropriate training of primary care clinicians [18].

We identified that challenges in selecting PROMs and concerns about relevance and reliability of PROMs in a primary care setting were barriers to their use. Similarly Dowrick et al. (2009) reported some GPs felt that PROMs do not adequate represent the dynamic nature of patients’ conditions or capture the complexity of symptoms [16]. Selection of appropriate PROMs for general practice has been debated [8, 19]. Condition and symptom specific PROMs present challenges given the diversity of conditions and symptoms seen in primary care and presence of multimorbidity. However, generic PROMs are often developed for long-term conditions or refer to a disease, which may not reflect all health problems in general practice [20]. Some PROMs have been developed specifically for primary care including the Patient Enablement Instrument [21] and the Primary Care Outcomes Questionnaire [22, 23]. However, choice of measure should be based on rationale for assessment. This was highlighted by GPs in our study who valued PROMs to aid clinical management and as screening/diagnostic tools.

### Implications for research and/ or practice

Lack of knowledge and training about PROMs were important barriers to their use. Systematic training and exposure to PROMs is necessary to motivate GPs to integrate PROMs into routine care and empower them to make informed decisions regarding when to use PROMs and which measures to select. However, even with appropriate training, organisational barriers need to be addressed, particularly integration within clinical systems to this end we have produced a systematic analysis of the factors affecting the current implementation of PROMs [12].

PROMs collected routinely in primary care could subsequently be used for audit and benchmarking; however, our research suggests that this should not be the primary purpose of PROM collection. Optimal implementation of PROMs into routine clinical practice requires a bottom-up approach driven by needs of primary care clinicians and patients. Our study was restricted to GPs, future research should explore experiences of other primary care healthcare providers, particularly practice nurses who conduct long-term conditions reviews, and patients.

## Conclusion

PROMs have a potential to aid clinical management and diagnosis in primary care. However, current use is fragmented and there is a lack of knowledge regarding what PROMs are available, when to use them and their evidence base. Implementation of PROMs in primary care requires integration with clinical systems, a bottom-up approach to PROM selection and system design involving meaningful consultation with patients and primary care clinicians and training/support for use.

## Supplementary information

**Additional file 1.** Topic guide for semi-structured interviews, particpant characteristics, and description of commonly used PROMs.

## Data Availability

The datasets used and/or analysed during the current study are available from the corresponding author on reasonable request.

## Abbreviations

GPs: General Practitioners
NHS: National Health Service
PROMs: Patient Reported Outcome Measures
QOF: Quality and Outcomes Framework
