# Supplementary material for: General practitioners’ views on use of patient reported outcome measures in primary care: a cross-sectional survey and qualitative study
Source: BMC Fam Pract. 2020 Jan 24;21:14. doi: 10.1186/s12875-019-1077-6 (PMC6979354; doi:10.1186/s12875-019-1077-6)
Supplement: Supplementary file 1 — Additional file 1. Topic guide for semi-structured interviews, particpant characteristics, and description of commonly used PROMs. [file 12875_2019_1077_MOESM1_ESM.docx]

**Topic guide for semi-structured interviews**

**Use of PROMs in care**

- For which reasons do you currently use PROMs?

*Prompts* – Screening, diagnosis, monitoring, shared decision making (care plans, end of life care), QOF

- What influences your choice on the Proms you use

*Prompts* –How do you hear about the PROMs that are available?; Is there a standard set of PROMs used across the practice?; Who recommends them (CCG, colleague, other)

- How frequently do you use PROMs?
- Which individuals use PROMs in your organisation?

*Prompts* – Clinical staff e.g. GPs, Nurse practitioners, HCAs, Community matron, Physician associate; Non-clinical staff – receptionists, administrators, practice managers; Patient participation group (in partnership with practice)

- How do you use the data which are gained from PROMs?

*Prompts* – How do they inform care? Evaluation of practice performance? Resource allocation? Who is involved and how?

**Future PROM use**

- Which areas/groups of patients (if any) do you think PROMs could be used more effectively?

*Prompts* - Multi-morbidity, Metric of care (patient perception), Carers, Part of discharge note? Other?

- Which factors would facilitate the effective use of PROMS?

*Prompts* - Integration with existing systems/processes, Integration with EHR
Automatically generated and distributed to patients based on algorithm or diagnosis, Utility and usability (Practical/useful/relevant), Recommended by trusted source

- What are the main barriers to increased use of PROMs?

*Prompts* - Time, Resource, Training, Patient activation

**Table S1: Participant characteristics**

| **ID** | **Gender** | **Years qualified** | **Clinical Commissioning Group** | **Region** |
| --- | --- | --- | --- | --- |
| GP1 | F | 29 | Merton | South East |
| GP2 | M | 10 | Southport and Formby | North West |
| GP3 | F | 17 | Northumberland | North East |
| GP4 | M | 12 | North Durham | North East |
| GP5 | F | 20 | South Tyneside | North East |
| GP6 | M | 12 | South Eastern Hampshire | South |
| GP7 | F | 33 | Dorset | South |
| GP8 | M | 24 | Oxfordshire | South East |
| GP9 | F | 11 | Newark and Sherwood | East Midlands |
| GP10 | F | 13 | Southampton City | South |
| GP11 | F | 11 | Greenwich | South East |
| GP12 | M | 4 | Birmingham and Solihull | West Midlands |
| GP13 | M | 10 | North East Essex | South East |
| GP14 | M | 13 | East and North Hertfordshire | South East |
| GP15 | M | 17 | Fylde and Wyre | North West |
| GP16 | M | 10 | Sheffield | North East |
| GP17 | M | 4 | Birmingham and Solihull | West Midlands |
| GP18 | M | 2 | Birmingham and Solihull | West Midlands |
| GP19 | M | 8 | Dudley | West Midlands |
| GP20 | F | 31 | Salford | North West |
| GP21 | F | 7 | Bristol, North Somerset And South Gloucestershire | South West |
| GP22 | M | 30 | South Cheshire | North West |
| GP23 | F | 8 | Sheffield | North East |
| GP24 | F | 9 | Cambridgeshire and Peterborough | South East |
| GP25 | M | 12 | Dudley | West Midlands |

**Table S2. Description of commonly used patient-reported outcome measures in primary care**

| Measure | Description | Measurement properties (as recorded on PROQOLID) |
| --- | --- | --- |
| EuroQol 5 Dimension (EQ-5D) | A 5-item PRO (utility) measure with a self-classifier and a visual  analogue scale (VAS) which can be used to value health states.^1^ The self-classifier includes 5 dimensions: (i) mobility (ii) self-care (iii) usual activities (iv) pain/discomfort (v) anxiety/depression. Recall period is today. Each dimension has 3 levels of severity (no problems, some problems, and severe problems) and it is possible to describe 243 health states between 0 (dead) and 1 (perfect health).^1^ Higher scores indicate better HRQOL. | *Reliability:*  Test-retest (Intraclass correlation coefficient) - 0.77 (EQ VAS score) and 0.89 (EQ-5D index score)  *Validity:*  Significant correlations between each WHO-5 domains and the EQ-5D-5L (p<0.001)  *Ability to detect change:*  Minimal Important Difference (MID), Mean MID = 0.074 (range -0.011 - 0.140). |
| Patient Health Questionnaire-9 (PHQ) - 9 | A 9-item PRO measure which scores each of the 9 DSM-IV criteria. It is the depression module of the original Primary Care Evaluation of Mental Disorders Patient Questionnaire (PRIME-MD). A 4-point Likert scale is used and items are scored as “0” (not at all) to “3” (nearly every day). Higher scores indicate worse  HRQOL.^2^ | *Reliability:*  Internal consistency (Cronbach's alpha) - 0.89 |
| Generalized Anxiety Disorder-7 (GAD-7) | A 7-item PRO measure designed to identify probable cases of Generalized Anxiety Disorder (GAD) and assess symptom severity in GAD.  Utilises a 4-point Likert scale. Items are scored as “0” (not at all) to “3” (nearly every day) and global scores range from 0-21. Higher score indicate higher severity of anxiety symptoms.^3^ | *Reliability:*  Internal consistency (Cronbach's alpha) - 0.92  Test-retest (Intraclass correlation coefficient) - 0.83  *Validity:*  Correlation of the GAD-7 with the Beck Anxiety Inventory, r=0.72 |
| Epworth Sleepiness scale (ESS) | An 8-item PRO measure for measuring a subject's usual level of daytime sleepiness or average sleep propensity. Utilises a 4-point Likert scale. Global scores range from 0-24. Higher scores indicate higher sleepiness.^4^ | *Reliability:*  Internal consistency (Cronbach's alpha) - 0.88  Test-retest (Pearson correlation coefficient) - 0.822; p<0.001  *Validity:*  Correlation between ESS scores and Respiratory Disturbance Index (RDI), (r=0.55, p<0.001) |
| Hospital Anxiety and Depression Scale (HADS) | A 14-item PRO measure to detect states of anxiety and depression.^5^ Anxiety domain (7 items) and depression domain (7 items). Utilises a 4-point Likert scale. Recall period is the past week. Scored by individual domain. Higher scores indicate worse HRQOL. | *Reliability:*  Internal consistency (Cronbach's alpha) - 0.93 for anxiety scale, 0.90 for the depression scale  *Validity:*  HAD-A with Clinical Anxiety Scale, r=0.67  HAD-D with Montgomery-Asberg Depression Rating Scale, r=0.77 |
| Modified Medical Research Council Dyspnea Scale (mMRC) | A 22-item composite measure (PRO and PerfO) to diagnose the chronic bronchitis.^6^ Dichotomous ‘Yes/No’ and open-ended questions. Recall period varies from ‘this morning’ to patient’s entire life depending on the item.^6^ Higher scores indicate higher severity. | Information not available on PROQOLID |
| Edinburgh Postnatal Depression Scale (EPDS) | A 10-item PRO measure to screen postnatal depression.^7^ Utilises a 4-point Likert scale. Recall period is in the past 7 days. Global scores range from 0-30. Higher scores indicate higher severity of depressive symptoms.^7^ | *Reliability:*  Internal consistency (Cronbach's alpha) - 0.87 |
| International Prostate Symptom Score (I-PSS) | An 8-item PRO measure to capture the severity of urinary symptoms related to benign prostatic hyperplasia.^8^ 6- to 7-point Likert scale. Recall period is over the past month (standard) or over the past week (acute). Scores for the symptoms dimension range from 0 to 35 and higher scores indicate more severe symptoms. Scores for the impact on QOL item range from 0 to 6 (can be transformed on a scale from 0 to 100) and higher scores indicate higher impact on HRQOL.^8^ | *Reliability:*  Internal consistency (Cronbach's alpha) - 0.86  Test-retest (Pearson product-moment coefficient), r=0.92    *Validity:*  Receiver Operating Characteristic (ROC) curve = 0.85±0.03  *Ability to detect change:*  Minimal Important Difference (MID) - 3 points |
| Oxford Hip Score (OHS) | A 12-item PRO measure to assess the outcomes of hip arthroplasty.^9^ Pain and function domains are combined in one composite scale.^9^ Recall period is past 4 weeks. Utilises a 5-point Likert scales ranging from 1 to 5. Global scores range from 0 to 48. Higher scores indicate least symptoms/problems. | *Reliability:*  Internal consistency (Cronbach's alpha) - 0.89  Test-retest (Bland Altman Coefficient of reliability) -7.27 |
| Oxford Knee Score (OKS) | A 12-item PRO measure to assess outcomes of knee arthroplasty.^10^ Recall period is past 4 weeks. Utilises a 5-point Likert scales ranging from 1 to 5. Global scores range from 0 to 48. Higher scores indicate better/less problems.^10^ | *Reliability:*  Internal consistency (Cronbach's alpha) - 0.93  Test-retest (Pearson coefficient), r=0.92  *Ability to detect change:* Effect size – 2.19 |
